# Supplementary material for: Analysis of PPARγ Signaling Activity in Psoriasis
Source: Int J Mol Sci. 2021 Aug 10;22(16):8603. doi: 10.3390/ijms22168603 (PMC8395241; doi:10.3390/ijms22168603)
Supplement: Supplementary file 1 [file ijms-22-08603-s001.zip › Supplemental materials_Analysis of PPARg signaling activity in psoriasis/Pathway models/Models images and html files/Interleukin-17 and interleukin-22 signaling in psoriasis/8103.html]

NF-kB family


# Functional Class NF-kB family

|  |  |
| --- | --- |
| URN | urn:agi-aopfc:0000156 |
| Total Entities | 8 |
| Connectivity | 19702 |
| Name | NF-kB family |
| Description | nuclear factor kappaB |
| ObjectType | Functional Class |
| Mol Function | Transcription factor |

---

|  |  |
| --- | --- |
| ChildConcepts | NFKB1 |
|  | NFKB1/RelA |
|  | NFKB2 |
|  | REL |
|  | cRel/RelA |
|  | RELA |
|  | RelA/RelB |
|  | RELB |

---

|  |  |
| --- | --- |
| Pathway | Osteoarthritis |
|  | MTHFR Activity Regulation |
|  | Melatonin Effects on Circadian Cycle |
|  | Fatty Acids Block TLR Signaling in Enteroendocrine Cell |
|  | Dopamine Receptors Signaling |
|  | Cortisol in Resolving Inflammation |
|  | Testicular AMH Production |
|  | Toll-like Receptors Act through MYD88-TIRAP Signaling |
|  | NOD-like Receptors |
|  | Toll-like Receptors Act through MYD88 Signaling |
|  | TLR4 Signaling in Leukocytes |
|  | T-Cell Dependent B-Cell Activation |
|  | T-Cell Independent B-Cell Activation |
|  | V(D)J Recombination Activation |
|  | Immunoglobulin Class-Switch Recombination Activation |
|  | Natural Killer Cell Activation through ITAM-Containing Receptors |
|  | Natural Killer Cell Activation through ITSM-Containing Receptors |
|  | Natural Killer Cell Activation |
|  | Peripheral T-Cell Tolerance |
|  | T-Cell Central Tolerance |
|  | CD8+ T-Cell Activation |
|  | Th1-Cell Differentiation |
|  | Treg-Cell Differentiation |
|  | Th2-Cell Differentiation |
|  | T-Cell Receptor Signaling |
|  | AHR Signaling in Treg and Dendritic Cells Function |
|  | Th17-Cell Differentiation |
|  | AHR Signaling in Tr1 Cells Function |
|  | CXC Chemokine Receptor Signaling |
|  | CC Chemokine Receptor Signaling |
|  | Plasmin Effects in Inflammation |
|  | Proplatelet Maturation |
|  | Bradykinin Effects in Inflammation |
|  | Toll-like Receptors in Sterile Inflammation |
|  | Toll-like Receptor Independent Sterile Inflammation |
|  | Necrosis |
|  | Lipoxin A4/FPR2-Related Neutrophil Depression |
|  | MC1R in anti-Inflammatory Signaling |
|  | Lipids Enhance Apoptotic Cell Engulfment and Reduce Inflammation |
|  | Prostaglandin E2 Receptor Signaling in Neurons |
|  | Leukotriene Effect on Vascular Endothelial Cell Response |
|  | Eicosanoids in Myeloid Cell Activation |
|  | Neutrophil Activation via LTB4/Polyisoprenyl Phosphate Signaling |
|  | Basophil Activation |
|  | Eosinophil Survival by Cytokine Signaling |
|  | Eosinophil Activation |
|  | Mast-Cell Activation without Degranulation through TNFSF8 Signaling |
|  | Mast-Cell Activation via IgE Signaling |
|  | Mast-Cell Activation without Degranulation |
|  | Mast-Cell Activation without Degranulation through IL33/IL1RL1 Signaling |
|  | Mast-Cell Activation without Degranulation through KIT/F2R Signaling |
|  | Mast-Cells De Novo Synthesized Mediators via IgE Independent Signaling |
|  | Mast-Cell Activation without Degranulation through IL1R1 and TLR Signaling |
|  | Macrophage Related Iron Uptake and Release |
|  | Macrophage M1 Lineage |
|  | CD16/CD14 Proinflammatory Monocyte Activation |
|  | AHR Signaling in M1 Macrophages Function |
|  | anti-Inflammatory Function of Macrophage M2 Lineage |
|  | AXL Receptor Inhibits Macrophages and Dendritic Cells Function |
|  | Neutrophil Recruitment and Priming |
|  | Neutrophil Activation via Adherence on Endothelial Cells |
|  | Negative Acute Phase Proteins Synthesis |
|  | Positive Acute Phase Proteins Synthesis |
|  | Vascular Endothelial Cell Activation by NO |
|  | Vascular Endothelial Cell Activation by Cytokines |
|  | Vascular Endothelial Cell Activation by Blood Coagulation Factors |
|  | Vascular Endothelial Cell Activation by Growth Factors |
|  | Vascular Endothelial Cell Permeability Activation |
|  | Adipokines Production by Adipocyte |
|  | Lipolysis Regulation in Adipocyte |
|  | Nociception Expression Targets Signaling |
|  | BDKRB1/2 -> Interleukins Production |
|  | DRD2 -> TRPC1 Transcription |
|  | TACR1 -> TNF/IL6/IL8 Production |
|  | IL1B Expression Targets -> Nociception |
|  | Melatonin in Cell Survival and Antioxidant Response |
|  | Apoptosis |
|  | SCF/FBXW7 Complex |
|  | Cardiomyocyte Dysfunction |
|  | Scavenger Receptor OLR1 in Inflammation Related Endothelial Dysfunction |
|  | Dendritic Cells Function in Atherosclerosis |
|  | RAGE/AGER and S100 Proteins in Cardiovascular Injury |
|  | Low-Density Lipoproteins and Chemokines in Atherosclerosis |
|  | Th1-Cells Activation and Proliferation in Atherosclerosis |
|  | Dilated Cardiomyopathy |
|  | Proteins Involved in Hypertrophic Cardiomyopathy |
|  | Cardiomyocyte Hypertrophy |
|  | Hypertrophic Cardiomyopathy |
|  | Proteins Involved in Hypoxia-Induced Pulmonary Hypertension |
|  | Endothelial Cell Dysfunction in Pulmonary Hypertension |
|  | Myocardial Remodeling |
|  | Myocardial Infarction |
|  | MAVS in Antiviral Innate Immune Response in Myocarditis |
|  | Lymphocyte Mediated Myocardial Injury in Myocarditis |
|  | NO Synthesis in Early Vasospasm |
|  | Inflammatory Reaction in Acne Vulgaris |
|  | Keratinocyte Proliferation in Acne Vulgaris |
|  | Telogen Maintenance in Androgenic Alopecia |
|  | Atopic Dermatitis Onset |
|  | Mast-Cells Activation in Atopic Dermatitis |
|  | Acute Phase in Atopic Dermatitis |
|  | Atopic Dermatitis |
|  | Melanoma |
|  | MITF as a Regulator of Melanoma Cell Development |
|  | IL17 Signaling in Psoriasis |
|  | IFN-gamma/TNF-alpha Mediated Cell Proliferation in Psoriasis |
|  | Dendritic Cells Function in Psoriasis |
|  | Melanocyte Role in Skin Aging |
|  | Apoptotic Keratinocytes Clearance Recession in Systemic Lupus Erythematosus |
|  | Dendritic Cell Activation in Systemic Lupus Erythematosis |
|  | Th0-Cell Aberrant Activation in Systemic Lupus Erythematosus |
|  | Th17-Cell Function in Systemic Lupus Erythematosus |
|  | B-Cell Activation in Systemic Lupus Erythematosus |
|  | Th2-Cell Function in Systemic Lupus Erythematosus |
|  | Acute Cytotoxic CD8+T-Cell Response against Melanocytes |
|  | T-Cell Cytotoxic Response against Melanocytes in Vitiligo |
|  | Humoral Immunity in Vitiligo |
|  | Treg-Cell Function in Diabetes Mellitus Type 1 |
|  | Natural Killer T-Cell Roles in Diabetes Mellitus Type 1 |
|  | non-Suppressive Treg-Cell in Diabetes Mellitus Type 1 |
|  | Plasmacytoid Dendritic Cell in Diabetes Mellitus Type 1 |
|  | TLR2 Signaling in Treg-Cell in Type 1 Diabetes (Animal Model) |
|  | Natural Killer Cell in Diabetes Mellitus Type 1 |
|  | Toll-like Receptors in beta-Cell |
|  | Macrophage Function in Diabetes Mellitus Type 1 |
|  | Peripheral Tolerance to Autoantigens Recession in Diabetes Mellitus Type 1 |
|  | TLR7 and TLR9 in Plasmacytoid Dendritic Cell |
|  | Conventional Dendritic Cell Role in Diabetes Mellitus Type 1 |
|  | Treg-Cell Activation in Diabetes Mellitus |
|  | Advanced Glycation End Products Pathway in Diabetic Microangiopathy |
|  | Protein Kinase C in Diabetic Microangiopathy |
|  | Peripheral Tissue Microangiopathy |
|  | Tubular Cell Dysfunction in Progressive Diabetic Nephropathy |
|  | Podocyte Dysfunction in Diabetic Nephropathy |
|  | Endothelial Cell Dysfunction in Early Diabetic Nephropathy |
|  | Microangiopathy in Diabetic Neuropathy |
|  | Proliferative Diabetic Retinopathy |
|  | AGE Related Insulin Resistance in Skeletal Muscles |
|  | Adipose Tissue Inflammation Provokes Insulin Resistance |
|  | Hepatic Stellate Cells in non-Alcoholic Fatty Liver Disease |
|  | Oxidation of Fatty Acid in non-Alcoholic Fatty Liver Disease |
|  | beta-Cell Destruction through Cytokines in Diabetes Mellitus |
|  | beta-Cell Recovery in Diabetes Mellitus |
|  | beta-Cell Destruction in Diabetes Mellitus Type 1 |
|  | Nitric Oxide Effects on beta-Cell |
|  | Immune Sustem Activation in Hashimoto's Thyroiditis |
|  | Hashimoto's Thyroiditis |
|  | RET1 Mutation in Hyperparathyroidism |
|  | Bone Remodeling in Hyperthyroidism |
|  | Immune System Activation in Hyperthyroidism |
|  | Thymic Follicular Hyperplasia |
|  | Fatty Acids in Hypertension and Atherosclerosis Development |
|  | Adipokines Production by Adipocyte Impaired in Obesity |
|  | Proteins Involved in Obesity |
|  | CD4+T-Cell Response in Celiac Disease |
|  | Metastatic Colorectal Cancer |
|  | Crohn's Disease |
|  | Paneth-Cell Function in Crohn's Disease |
|  | Macrophage Dysfunction |
|  | Dendritic Cell Dysfunction in Crohn's disease |
|  | OipA Signaling in Helicobacter Pylori Infection |
|  | CagA Phosphorylation Independent Signaling |
|  | Dendritic Cell Function in Ulcerative Colitis |
|  | Epithelial Cell in the Innate Immune Response in Ulcerative Colitis |
|  | Leukocyte Migration toward the Endothelial Cell in Ulcerative Colitis |
|  | Nicotine anti-Inflammatory Effect in Ulcerative Colitis |
|  | Th2-Cell Function in Ulcerative Colitis |
|  | T-Cell Cytotoxic Response in Ulcerative Colitis |
|  | Proteins Involved in Acute Myeloid Leukemia |
|  | Apoptosis Block in Acute Myeloid Leukemia Cell |
|  | Acute Myeloid Leukemia |
|  | Antiphospholipid Antibodies in Endothelial Cell |
|  | B-Cell Acute Lymphoblastic Leukemia |
|  | Proteins Involved in B-Cell Chronic Lymphocytic Leukemia |
|  | B-Cell Chronic Lymphocytic Leukemia |
|  | Chronic Myeloid Leukemia |
|  | Proteins Involved in Chronic Myeloid Leukemia |
|  | Diffuse Large-B-Cell Lymphoma |
|  | Diffuse Large-B-Cell Lymphoma ABC Subtype |
|  | Proteins Involved in Diffuse Large-B-Cell Lymphoma |
|  | Primary Mediastinal B-cell Lymphoma (PMBL) Subtype |
|  | Follicular Lymphoma |
|  | Hodgkin and Reed-Sternberg Cells in Hodgkin Lymphoma |
|  | Proteins Involved in Hodgkin Lymphoma |
|  | Hodgkin Lymphoma |
|  | Proteins Involved in Mantle Cell Lymphoma |
|  | Mantle Cell Lymphoma |
|  | Apoptosis of Mantle Cell Deregulation |
|  | mTOR/NF-kB/BCR Signaling Disregulation in Mantle Cell Lymphoma |
|  | Mucosa-Associated Lymphoid Tissue (MALT) Lymphoma |
|  | Splenic Marginal Zone Lymphoma |
|  | Proteins Involved in Marginal Zone Lymphoma |
|  | Osteoclast Activation in Multiple Myeloma |
|  | IL6/IGF1/VEGFA Signaling in Multiple Myeloma |
|  | Multiple Myeloma |
|  | Proteins Involved in Multiple Myeloma |
|  | Antiphospholipid Antibodies and Endothelial Cell Interaction |
|  | Proteins Involved in T-Cell Acute Lymphoblastic Leukemia |
|  | T-Cell Acute Lymphoblastic Leukemia |
|  | SERPINA1 Associated Liver Damage |
|  | Eosinophil Survival in Asthma |
|  | Il17 Signaling Related Neutrophilia in Asthma |
|  | IL33 Signaling Related Eosinophilia in Asthma |
|  | TSLP Signaling in Bronchial Epithelial Cell in Asthma |
|  | IL17F Signaling in Bronchial Epithelial Cell in Asthma |
|  | IgE Induces Airway Smooth Muscle Cell Proliferation |
|  | Goblet-Cell Related Mucus Secretion in Asthma |
|  | Club-Cell Role in Asthma |
|  | Neutrophil Degranulation via FPR1/IL8 |
|  | Basophil Activation in Asthma |
|  | Mast-Cell Activation in Asthma |
|  | Mast-Cell Related Chemokines Signaling in Asthma |
|  | Antigen-Presenting Cell Role in Asthma |
|  | B-Cell Mediated IgE Production in Asthma |
|  | Th17-Cell Differentiation in Asthma |
|  | Th2-Cell Response in Asthma |
|  | Mucin Production in Goblet Airway Epithelial Cells |
|  | CFTR Facilitates Oxidative Stress in Airway Epithelium in CF |
|  | Airway Epithelial Cell Dysfunction |
|  | CFTR Expression in Epithelial Cells (Class I Mutations) |
|  | CFTR Related Ion-Channel Dysfunction in Cystic Fibrosis Airway Epithelium (Class III Mutations) |
|  | Osteoclast Activation in Gout |
|  | Neutrophil Recruitment in Sinovium in Gout |
|  | Monocytes Function in Gout |
|  | Hyper IgM Syndrome |
|  | TNF and IL1B Induce Metalloproteinase Synthesis in Osteoarthritis |
|  | Apoptosis of Chondrocyte |
|  | IL1B Induced Arthralgia |
|  | Th17-Cell and Th1 Immune Responsein Psoriatic Arthritis |
|  | Synovial Fibroblast Activation in Psoriatic Arthritis |
|  | Keratinocyte Activation in Psoriatic Arthritis |
|  | PTPN22 Role in Rheumatoid Arthritis |
|  | Osteoclast Activation in Rheumatoid Arthritis |
|  | Synovial Fibroblast Activation by Citokines in Rheumatoid Arthritis |
|  | Apoptosis Block in Synovial Fibroblast |
|  | TLR2 Induced Synovial Fibroblast Activation in Rheumatoid Arthritis |
|  | Autocrine Cytokine/Chemokine Loops in Systemic Scleroderma |
|  | B-Cells Function in Systemic Scleroderma |
|  | Th2-Cells Function in Systemic Scleroderma |
|  | Skin Fibrosis |
|  | CD4+ T-Cell Function Decline in HIV |
|  | Apoptosis Block in Infected Cells |
|  | CD4+ T-Cell Death in HIV |
|  | Toll-like Receptors (TLR) in Periodontitis |
|  | Diabetes Induced Periodontitis |
|  | IL1 Receptor Antagonist (IL1RN) Deficiency (DIRA) |
|  | PAPA Syndrome |
|  | TNF Receptor Associated Periodic Syndrome |
|  | Mevalonate Kinase Deficiency |
|  | Blau Syndrome |
|  | IL36 Receptor Antagonist (IL1F5) Deficiency (DITRA) |
|  | Cryopyrin (NLPR3) Associated Periodic Syndromes |
|  | Familial Cold Autoinflammatory Syndrome 2 |
|  | Familial Mediterranean Fever |
|  | Majeed Syndrome |
|  | Glutamate Toxiicity Effects on Striatum Medium Spiny Neurons in Hantington's Disease |
|  | NF-kB Signaling Activation in Huntington Disease |
|  | Proteins Involved in Huntington Disease |
|  | IL6 Promotes Inflammation in Muscular Dystrophies |
|  | IL6 Overexpression After Contraction in Skeletal Muscles in Muscular Dystrophies |
|  | Ca2+ Overload in Muscular Dystrophies |
|  | Muscular Dystrophies |
|  | Dystrophin Glycoprotein Complex Signaling in Muscular Dystrophies |
|  | Osteoclasts Function in Osteopetrosis |
|  | Osteoclast Activation in Postmenopause |
|  | Microglia Activation in Alzheimer's Disease |
|  | Proteins Involved in Epilepsy |
|  | Ketogenic Diet Mechanism (Hypothesis) |
|  | Blood-Brain Barrier Disruption in Epileptiform Disorders |
|  | HMGB1 and IL1B in Neuroinflammation |
|  | Glioblastoma, Primary |
|  | Proteins Involved in Glioblastoma |
|  | Glioblastoma, Secondary |
|  | Astrocytoma |
|  | Proteins Involved in Astrocytoma |
|  | Proteins Involved in Glioma |
|  | Vascular Endothelial Cell Vasodilation in Brain |
|  | Postsynaptic Neuron Activation |
|  | nNOS (NOS1) and iNOS (NOS2) Activation via Glutamate |
|  | Growth Factor Signaling in Neuroblastoma |
|  | Neuroblastoma |
|  | Microglia Activation in Parkinson's Disease |
|  | Glutamate Role in Parkinson's Disease |
|  | Glutamate Mediated Excitotoxicity in Amyotrophic Lateral Sclerosis |
|  | Microglia and Motor Neuron Interaction Dysregulation |
|  | Crystallin alpha B (CRYAB) Suppresses Multiple Sclerosis |
|  | MIR155 Role in Th17-Cell in Multiple Sclerosis |
|  | Breast Cancer |
|  | Coagulation Factors and PAR1/2 Receptors (F2R/F2RL1) in Endometriosis |
|  | Dioxin Role in Endometriosis |
|  | MIF Role in Cholesteatoma |
|  | EGF/cyclinD1 Signaling in Acquired Middle Ear Cholesteatoma |
|  | HMGB1/RAGE Signaling in Middle Ear Cholesteatoma |
|  | Subepithelial Fibroblasts in Middle Ear Cholesteatoma |
|  | Cholesteatoma |
|  | HPV Infection and Cancer |
|  | HPV E6 and E7 Induced Disruption of TNFR/NF-kB Signaling in Keratinocytes |
|  | Androgen Receptor/FKBP5 Signaling |
|  | Prostate Cancer |
|  | Oxidative Stress, All-Trans-Retinal and Lipofuscin Toxicity in AMD |
|  | TLR3 Role in Retinal Pigment Epithelial Cell |
|  | ER Stress in Macular Degeneration |
|  | EPHA2 Mutations in Cataract |
|  | OPTN Effects on NF-kB Signaling in Glaucoma |
|  | Glutamate Mediated Excitotoxicity in Glaucoma |
|  | OPTN Associated Glaucoma |
|  | Neurotrophic Factor Deprivation in Retinal Ganglion Cell Death |
|  | Proteins Involved in Hearing Loss |
|  | Cochlear Inflammation (Noise-Induced) |
|  | Cytokines Trigger Otitis Media |
|  | Immune System Role in Otitis Media |
|  | Alveolar Macrophage Negative Regulation Declined in COPD |
|  | Alveolar Macrophages Dysfunction in COPD |
|  | Proteins Involved in Chronic Obstructive Pulmonary Disease |
|  | CAV1 Dependent Senescence of Lung Fibroblasts |
|  | T-Cell Cytotoxic Activation |
|  | Alveolar Epithelial Cell Dysfunction in COPD |
|  | Mucin Hyperproduction in Goblet and Mucous Cells |
|  | Proteins with Altered Expression in Cystic Fibrosis |
|  | Advanced Glycation End Products in Diabetes or Age Related Erectile Dysfunction |
|  | Plasmin Role in Glomerulonephritis |
|  | Endothelial Cell Dysfunction in Glomerulonephritis |
|  | Podocyte Dysfunction in Glomerulonephritis |
|  | Endothelial Cell Dysfunction in Pyelonephritis |
|  | Cryptorchidism Molecular Mechanism (Hypothesis) |
|  | Sertoli Cells Dysfunction Causes Male Infertility |
|  | Ammonia Effects on Brain Cells |
|  | CD157/ITGB2 Signaling in Myeloid Cell |
|  | CD38/CD3 -> JUN/FOS/NF-kB Signaling in T-cell Proliferation |
|  | CD38/CD19 -> JUN/FOS/NF-kB Signaling in B-cell Proliferation |
|  | S100P Expression Target |
|  | HMGB1 Expression Targets |
|  | TTR Expression Targets |
|  | S100B Expression Targets |
|  | Dopamine/Gi Expression Targets |
|  | B-Cell Receptor -> NF-kB Signaling |
|  | TGFBR3 -> ARRB2 Signaling |
|  | TLR -> ARRB2/NF-kB Signaling |
|  | CD19 Expression Targets |
|  | CD81 -> AP-1 Expression Targets |
|  | gamma Globulins Expression Targets |
|  | CD22 -> NF-kB Expression Targets |
|  | CD19/CR2 Expression Targets |
|  | CD72 -> STAT Expression Target |
|  | CD8 -> STAT Expression Targets |
|  | CD80 -> STAT Expression Targets |
|  | CD86 -> STAT Expression Targets |
|  | CSF2 -> NF-kB Expression Targets |
|  | Complement Component GPCRs Signaling |
|  | Doxorubicin Induced Cardiotoxicity |
|  | Clozapine Induced Granulocytopenia |
|  | Cyclosporine Induced Nephrotoxicity |
|  | Cocaine Induced Hepatotoxicity |
|  | Erythropoietin -> NF-kB Expression Targets |
|  | PECAM1 Expression Targets |
|  | F2 -> STAT1/NF-kB Expression Targets |
|  | PLG -> STAT1/NF-kB Expression Targets |
|  | GH1/GHR -> NF-kB/ELK/SRF/MYC Expression Targets |
|  | CSH1/GHR Expression Targets |
|  | TAM Receptor Signaling Activation in Cancer |
|  | Asbestosis- and Silicosis-Associated Inflammation Provokes Cancer Development |
|  | TGFB Signaling activation by Blocking of Tumor Suppressors |
|  | Integrins in Cancer Cell Motility, Invasion and Survival |
|  | Epithelial to Mesenchymal Transition in Cancer: Overview |
|  | Cancer Cells Inhibit Adipocyte Differentiation |
|  | Skeletal Muscle Wasting in Cancer Cachexia |
|  | Lipogenesis/Lipolysis Activation in Cancer Cells and Cancer-Associated Adipocytes |
|  | Treg Cells Promote Immunosuppression in Cancer Immune Escape |
|  | IDO1 in Cancer Immune Escape |
|  | Effector T-cell Inactivation in Cancer Immune Escape |
|  | CD46/CD55/CD59 Inhibit Complement Mediated Lysis of Cancer Cells |
|  | DNMT and MBD Families Activation in DNA Methylation in Cancer |
|  | CD19 -> NF-kB Signaling |
|  | AGER -> NF-kB Signaling |
|  | FibronectinR -> NF-kB Signaling |
|  | MacrophageR -> CEBPB -> NF-kB Signaling |
|  | Fibronectin Expression Targets |
|  | ICAM1 Expression Targets |
|  | Fibrinogen Expression Targets |
|  | IFNLR1-> STAT/NFKB Signaling |
|  | IL7R -> FOXO/NF-kB Signaling |
|  | IL15R -> NF-kB/NFATC Signaling |
|  | IL23R -> STAT3/NF-kB Signaling |
|  | IL1R -> NF-kB Signaling |
|  | IL12R -> NF-kB/NFATC Signaling |
|  | IL7 Expression Targets |
|  | IL15 Expression Targets |
|  | IL12B Expression Targets |
|  | IL16 -> NF-kB Expression Targets |
|  | PAF/Gq -> NF-kB Expression Targets |
|  | MTNR1 Signaling |
|  | NGF -> SMAD3/NF-kB Expression Targets |
|  | BDNF Expression Targets |
|  | NTF3 Expression Targets |
|  | NTF4 Expression Targets |
|  | NOTCH -> NF-kB Signaling |
|  | DLL4 Expression Targets |
|  | ADAM17 Expression Targets |
|  | JAG1 Expression Targets |
|  | DLL1 Expression Targets |
|  | NOTCH Expression Targets |
|  | Adenosine Expression Targets |
|  | PRL/GHR -> NF/kB/ELK/SRF/MYC Expression Targets |
|  | PDCD1 -> NF-kB Expression Targets |
|  | DDR1 -> NF-kB Signaling |
|  | Collagen -> NF-kB Expression Targets |
|  | TNF-alpha/TNFRSF1A Signaling |
|  | MERTK Signaling |
|  | TGF-beta Signaling |
|  | HIF1 Signaling |
|  | AMPK Signaling |
|  | BMPR2 Signaling |
|  | AXL Receptor Tyrosine Kinase Signaling |
|  | TNF-alpha/TNFRSF1B Signaling |
|  | KRAS Signaling |
|  | TCR -> NF-kB Expression Targets |
|  | T-Cell Receptor -> NF-kB Signaling |
|  | FASLG Expression Targets |
|  | TNF -> NF-kB Expression Targets |
|  | TNFSF10 Expression Targets |
|  | LTA Expression Targets |
|  | TNFSF14 Expression Targets |
|  | CD40LG -> NF-kB/ELK/SRF -> CREB/NFATC Expression Targets |
|  | TNFSF13 Expression Targets |
|  | TNFSF13B Expression Targets |
|  | EDA Expression Targets |
|  | EctodysplasinR -> NF-kB Signaling |
|  | NGFR -> NF-kB Signaling |
|  | TNFR -> NF-kB Signaling |
|  | TLR3 Expression Targets |
|  | TLR5 Expression Targets |
|  | TLR7 Expression Targets |
|  | TLR1 -> 2/6 Expression Targets |
|  | IL1B Expression Targets |
|  | TLR4 -> NF-kB/IRF Expression Targets |
|  | IL1A Expression Targets |
|  | TLR9 Expression Targets |
|  | TLR4/5/7/9 -> NF-kB Signaling |
|  | TLR1/2/6 -> NF-kB Signaling |
|  | TLR3 -> NF-kB Signaling |
|  | ROS in Triggering Vascular Inflammation |
|  | ER Stress (Unfolded Protein Response) |
|  | Glutamate Mediated Neurotoxicity |
|  | Mitochondrial Damage Induced by Hypoxia |
|  | Ca2+ Cytosolic Overload |
|  | ace2 |
|  | New Pathway (2) |
|  | cypa |
|  | PPAR Psoriasis |
|  | prarg negative regulators, ps-positive |
|  | prarg neg,ukn expres targets, ps-positive |
|  | Neighbors of NF-kB family |
|  | Model of PPARG signaling in psoriasis |
|  | PPARG negative regulators and targets |
|  | Model of PPARG related pathways in psoriasis (short version) |
|  | PPARs and neuroinflamation |
|  | non-proteinal partners of SARSs |
|  | BCL6-TP53-BCL2 |
|  | dsRNA induces innate immunity and INFs in alveolar cells |
|  | Innate immunity in Leukocytes |
|  | AGER feed forward loop in innate immunity |
|  | Dendritic Cell Activation in COVID19 |
|  | 1\_Inflammation in keratinocytes in the pathology of acne |
|  | 2\_Acute phase initiation in atopic dermatitis |
|  | 2\_Interleukin-17 and interleukin-22 signaling in psoriasis |
|  | 1\_The onset of atopic dermatitis |
|  | 1\_TNF and IL1B provoke ECM degradation in osteoarthritis |
|  | 1\_Osteoclast-mediated bone resorption in postmenopausal women |
|  | 2\_Chondrocyte death in osteoarthritis |
|  | 2\_The role of endothelial cells in pyelonephritis |
|  | 2\_Podocyte dysfunction in glomerulonephritis |
|  | 3\_Role of neurotrophic factor deprivation in retinal ganglion cell death |
|  | 2\_Oxidative stress, all-trans-retinal, and lipofuscin toxicity in agerelated macular degeneration |
|  | 3\_Endoplasmic reticulum stress in age-related macular degeneration |
|  | 1\_Low-density lipoprotein internalization and the accumulation of lipids in atherosclerosis |
|  | 2\_Oxidized LDL causes endothelial dysfunction in atherosclerosis |
|  | 1\_Endothelial cells stimulate vasoconstriction and thrombosis in pulmonary hypertension |
|  | 2\_Cardiomyocyte hypertrophy |
|  | 3\_Cardiomyocyte dysfunction unrelated to cell junctions in ARVD |
|  | 3\_Role of alveolar macrophages in pulmonary tissue destruction in COPD |
|  | 1\_Th2 cell response in asthma |
|  | 3\_2\_Regulation of airway surface liquid (ASL) thickness |
|  | 3\_1\_Mucus accumulation in asthma |
|  | 2\_2\_Eosinophilia and neutrophilia in asthma: eosinophil apoptosis |
|  | 4\_2\_Airway smooth muscle cells proliferation and airway tissue remodeling |
|  | 4\_Mucin hyperproduction in goblet and mucous cells in COPD |
|  | 2\_Paneth cell dysfunction in Crohn's disease |
|  | 1\_Defects in response to pathogens in the gut promotes inflammation in Crohn's disease |
|  | 3\_Autophagy dysfunction in Crohn's disease |
|  | 3\_Leukocyte migration toward the endothelial cells in the intestine microvasculature |
|  | 2\_Intestine epithelial cells drive immunological responses in ulcerative colitis |
|  | 1\_Intestine epithelial cell dysfunction in ulcerative colitis |
|  | 2\_Lipotoxicity in NAFLD |
|  | 1\_CD4+ T cell and epithelial cell response in celiac disease |
|  | 1\_HIV entrance to the host cell and viral reproduction |
|  | 5\_Human papilloma virus E6 and E7 proteins promote epithelial cell proliferation |
|  | 2\_HIV - induced depletion of CD4+ T-cells |
|  | 3\_1\_HIV latency and low level of viral production |
|  | 3\_2\_HIV postintegration latency |
|  | 5\_The rationale for the treatment of anemia of chronic disease with erythropoietin |
|  | 4\_2\_Insulin resistance: inflammation-related insulin resistance |
|  | 3\_2\_Beta-cell proliferation and compensation: neogenesis/dedifferentiation |
|  | 4\_3\_Insulin resistance: FFA-related insulin resistance |
|  | 1\_CFTR expression and degradation in epithelial cells in cystic fibrosis (class I-II mutations) |
|  | 2\_SERPINA1-associated liver damage |
|  | 1\_Antigen-presenting cells promote islet dysfunction and immune system activation in T1D |
|  | 3\_Islet beta-cell destruction in T1D |
|  | 1\_Microangiopathy in diabetic neuropathy |
|  | 2\_Neuron and Schwann cell death |
|  | 1\_Glutamate and calcium excitotoxicity of motor neurons in ALS |
|  | 1\_1\_Glutamate overload enforced prostaglandins and NO synthesis in neurons |
|  | Figure 4\_Diabetes mellitus Type 1: conventional dendritic cell |
|  | Model of PPARG signaling in psoriais (tested) |
|  | Interleukin-17 and interleukin-22 signaling in psoriasis |

---

|  |  |
| --- | --- |
| Group | non protein partners |

---

|  |  |
| --- | --- |
| MedScan ID | 12000005 |

---

|  |  |
| --- | --- |
| Alias | NF-kappabeta |
|  | NFkappaB |
|  | nuclear factor kB |
|  | nuclear factor NF kappa B |
|  | NF-kB |
|  | nuclear factor kappa-light-chain-enhancer of activated B-cell |
|  | nuclear factor B |
|  | nuclear factor k-light-chain-enhancer of activated B-cell |
|  | NF-(k)B |
|  | immunoglobulin enhancer binding protein |
|  | nuclear factor kappaB |
|  | NF-kappaB |
|  | nuclear factor k-B |
|  | nuclear factor NFkappaB |
|  | NFkB |
|  | NF kappa B |
|  | NF{kappa |
|  | NFkappa B |
|  | Nuclear factor (NF) kappaB |
|  | Rel |
|  | N-kappa B |

---

|  |  |
| --- | --- |
| InChIKey | YMFNPBSZFWXMAD-UHFFFAOYSA-N |

---

|  |  |
| --- | --- |
| Cell Localization | Nucleus |

---

|  |  |
| --- | --- |
| LocatorString | pmftq:oncomed |
|  | pmquery:curcumin+prostate+cancer |
|  | digtext:C:\Users\Ariadne\Customers\Elan\Elan weekly report from literature condensed.txt |
|  | pmquery:curcumin prostate cancer |

---
